# Rush Medical College

**Published:** 1875-03-01

**Authors:** 


### RUSH MEDICAL COLLEGE.

THE thirty-second annual commencement of Rush Medical College was held at Central Hall, corner of Wabash avenue and Twenty-Second street. There was a very large attendance of the friends of the graduating class of 1875.

The exercises opened with prayer, after which Prof. J. W. Freer made a brief speech to the graduates, in which he gave them some fatherly advice, and hoped that their worldly career would be as prosperous as their collegiate experience had been pleasant.

The valedictory, which abounded with the pathetic eloquence characteristic of such efforts, was gracefully delivered by Dr. Henry Fritcher.

Prof. Henry M. Lyman then delivered an address, the subject of which was Count Rumford (once Benjamin Thompson), a Massachusetts man who was a Tory in principle, and who went to Europe, where he became a soldier, and subsequently a chemist. In Germany, his great merit won him a Countship—his title being taken from the American town in which he had spent his early days. To Thompson might be ascribed most of the modern improvements in kitchen ranges, furnaces, stoves, and many other things which are concomitants of a high state of civilization. He combined in himself three great essentials to success: energy, culture, and opportunity. He never missed a chance in life, and so made his mark.

The graduating class could only hope for professional success by hard work and the steady development of the talents that were in them.

Diplomas of medicine and surgery were then handed to the seventy-three graduates.

Dr. Ernest Sedgwick, on behalf of the graduating class, presented Prof. Joseph P. Ross with an elegant gold-headed cane, and an autograph album containing the signatures of all the class—a very beautiful present.

The Professor replied in fitting terms, and, after benediction, the proceedings terminated.

